# Ship roll motion prediction based on *ℓ*_1_ regularized extreme learning machine

**DOI:** 10.1371/journal.pone.0206476

**Published:** 2018-10-30

**Authors:** Binglei Guan, Wei Yang, Zhibin Wang, Yinggan Tang

**Affiliations:** 1 Logistics Engineering College, Shanghai Maritime University, Shanghai, 200135, China; 2 School of Electronic & Information Engineering, Ningbo University of Technology, Ningbo, Zhejiang, 315016, China; 3 State GRID Quzhou Power Supply Company, No. 6, XinHe road, Quzhou, 253000, Zhejiang, China; 4 Institute of Electrical Engineering, Yanshan University, Qinhuangdao, Hebei, 066004, China; Beijing University of Posts and Telecommunications, CHINA

## Abstract

In this paper, a new method is proposed for prediction of ship roll motion based on extreme learning machine (ELM). To improve the prediction accuracy and avoid over or under fitting, two techniques are adopted to select the appropriate structure of ELM. First, the inputs of the ELM are selected from the roll motion time series using Lipschitz quotient method. Second, the number of hidden layer nodes is determined via *ℓ*_1_ regularized technique. Finally, the *ℓ*_1_ regularized ELM is solved by least angle regression (LAR) algorithm. The effectiveness of the proposed method is demonstrated by ship roll motion prediction experiments based on the real measured ship roll motion time series.

## Introduction

Roll motion is one of the important motion modes for ship navigating in sea, which is caused by external environmental factors such as strong wind, waves and currents. Ship’s roll motion is undesirable especially under the condition of rough sea because it is harmful for ship’s stability, affects the safety of crew and cargos and gives rise to working inefficiency of seafarers. Therefore, ship roll motion prediction is very necessary because prediction information can give operator sufficient time to avoid serious events. However, ship roll motion prediction is a difficult problem because the dynamics of ship’s roll motion is a complex nonlinear system with time varying characteristics [1]. Moreover, roll motion is also coupled with and affected by other motion modes such as heave and pitch. So, it is hard to establish a precise model to predict ship’s roll motion.

Many researchers had put attention to ship roll motion prediction and put forward many prediction methods. Most of these prediction methods are based on time series analysis. In [2], a minor component analysis (MCA) was proposed to predict ship motion. Since the ship motion is a nonlinear processes, the nonlinear time series analysis method, neural network (NN) [3, 4] for example, is suitable to establish a prediction model. Yin et al. established radial basis function (RBF) neural networks to predict ship roll motion, where the structure and parameters of RBF network were adjusted online via sequential learning algorithm [5]. Since the ship motion is complex and time varying, wavelet analysis is a time-frequency signal analysis method, which can capture the changes of signals. Observing this, a ship roll motion prediction method based on wavelet network was proposed in [6], the wavelet network was adjusted through a coarse to fine process. While in [7], the ship roll motion time series data is first decomposed into different subbands, and then the subbands were used as inputs to train a variable RBF network, which is finally used as predictor of ship roll motion.

Extreme learning machine (ELM) is a new algorithm to train single hidden layer feed-forward neural networks (SLFNs) proposed by Huang in [8]. ELM transforms the training of SLFNs to a standard least square problem by randomly choosing the input weights and hidden bias, and thus is more efficient than conventional training algorithms in terms of training speed and computation efficiency. In [9], a ship roll motion predictor based on ELM was proposed. In [10], an improved OS-ELM was proposed to predict ship roll motion, where the number of nodes in hidden layer is determined using Akaike information criterion (AIC). In [11], a new ship roll motion prediction method was proposed by combining grey theory and OS-ELM, where the original time series was firstly processed to obtain a new accumulated time series by using the accumulated generation operation (AGO) in grey theory, and then, the mapping relationship between the accumulated time series and its prediction was built using OS-ELM. The actual prediction is obtained by the inverse accumulated generation operation (IAGO) performed on the predicted accumulated time series through OS-ELM.

For time series prediction based on neural network, determining the input variables is an important problem because it greatly affects the prediction accuracy. Moreover, determining the number of hidden nodes for neural networks is also an important problem. If large number of nodes are selected, it is possible to occur over-fitting. On the contrary, if small number of nodes are selected, under-fitting may occurs. Actually, for neural network based time series prediction, the above two problems are structure selection of neural networks. In the literatures related to ship roll motion prediction, the above two problems are less addressed simultaneously. In [5, 11, 12], sequential learning algorithm was adopted to obtain a variable structure RBF neural network for ship roll motion prediction, in which the number of hidden nodes is determined real time. However, the input variable selection wan not considered.

To overcome the above drawbacks, in this paper, a new prediction method for ship roll motion based on *ℓ*_1_-regularized ELM is proposed. In the proposed method, ELM is used as prediction model. The main contributions of this paper include two aspects. First, the predictors, namely the input variables and its number of prediction model, are determined from the view of function continuity, which is characterized by Lipschitz quotients. The proposed approach is different from phase space reconstruction method, where a fixed embedded dimension and time lag are assumed. Second, a *ℓ*_1_-regularized technique is utilized to select the node number of hidden layer of ELM. The training and structure determination of ELM are fulfilled simultaneously.

The rest of this paper is organized as follows. The principle of Lipschitz quotients is briefly reviewed In section 1. The *ℓ*_1_ regularized ELM is introduced in section 2. Section 1 presents the roll motion prediction process using *ℓ*_1_ regularized ELM. Finally, the simulated prediction results are presented in section.

## Lipschitz quotients

Originally, Lipschitz quotients is a ratio of two distances in Euclidean space. In [13], He and Asada adopted it to identify the orders of nonlinear dynamic system. More specifically, Lipschitz quotient was used as a measure if a variable is missed in a nonlinear function or is redundantly added in the function based on the continuity of the nonlinear function. In this paper, it is utilized to determine the appropriated inputs for time series prediction.

Considering a nonlinear function as

$$\begin{array}{c} y=f(x_{1},x_{2},\ldots ,x_{n}), \end{array}$$ (1)

where *n* is the number of input variables. For the sake of convenience, denote **x** = [*x*_1_, *x*_2_, ⋯, *x*_*n*_]^T^. Here, we pay our attention to the selection of input variables in reconstruction of function *f* from input-output pairs (**x**(*i*), *y*(*i*)), (*i* = 1, 2, …, *N*). If function *f* is continuous, its Lipschitz quotient *q*_*i*,*j*_, which is defined as

$$\begin{array}{c} q_{i,j}=\frac{\left|y(i)-y(j)\right|}{\left|x(i)-x(j)\right|},i\neq j, \end{array}$$ (2)

is bounded. In Eq (2), |**a** − **b**| represents the Euclidean distance between two points **a** and **b**. For function *f* in (1) with *n* input variables, its Lipschitz quotient $q_{i,j}^{\left(n\right)}$ can be calculated by extending Eq (2) as

$$\begin{array}{c} q_{i,j}^{\left(n\right)}=\frac{\left|y(i)-y(j)\right|}{\sqrt{{\left(x_{1}\left(i\right)-x_{1}\left(j\right)\right)}^{2}+\cdots +{\left(x_{n}\left(i\right)-x_{n}\left(j\right)\right)}^{2}}}, \end{array}$$ (3)

where the superscript *n* in $q_{i,j}^{\left(n\right)}$ denotes the correct number of input variables in (1). He and Asada revealed in [13] that if an input variable, *x*_*n*_ for example, is missed in reconstruction of *f*, its Lipschitz quotient $q_{i,j}^{\left(n-1\right)}$ will be extreme large or is larger than $q_{i,j}^{\left(n\right)}$, which relies on *x*_*n*_ is independent of other variables *x*_1_, *x*_2_, ⋯, *x*_*n*−1_ or not. On the other hand, if two or more redundant input variables are included in the reconstruction of *f*, its Lipschitz quotient $q_{i,j}^{\left(n+1\right)}$ will be very close to $q_{i,j}^{\left(n\right)}$ [13]. From the above findings, Lipschitz quotient can be used to select or determine the optimal number of input variables for reconstruction of *f*. In practice, there may be noise in input or output variables, the Lipschitz quotient maybe incorrect. To avoid the impact of measurement noise, a modified index as

$$\begin{array}{c} q^{\left(n\right)}={\left(\prod_{i=1}^{p}\sqrt{n}q^{\left(n\right)}\left(i\right)\right)}^{\frac{1}{p}}, \end{array}$$ (4)

are suggested in [13] for variable selection or order identification. In (4), *q*^(*n*)^(*i*) is the *i*-th largest Lipschitz quotient among all $q_{i,j}^{\left(n\right)}$ and *p* is a positive number usually selected to be *p* ∈ [0.01*N*, 0.02*N*]. In practical application, a stop criterion defined as

$$\begin{array}{c} \frac{|q^{\left(n+1\right)}-q^{\left(n\right)}|}{\max(1,|q^{\left(n\right)}|)}<\varepsilon , \end{array}$$ (5)

is used to terminate the algorithm, where the threshold *ε* = 0.1 is suggested in [13].

## *ℓ*_1_-regularized extreme learning machine (ELM)

### Basics of ELM

ELM was proposed by Prof. Huang in [8] to train a single hidden layer feedforward neural network (SFLN), aiming at simplifying and speeding up Ó the training process of SFLN. Different from traditional training methods, the input weights and bias in ELM are randomly initialized, and only the output weights are calculated analytically using Moore–Penrose (M-P) generalized inverse.

**Fig 1:**
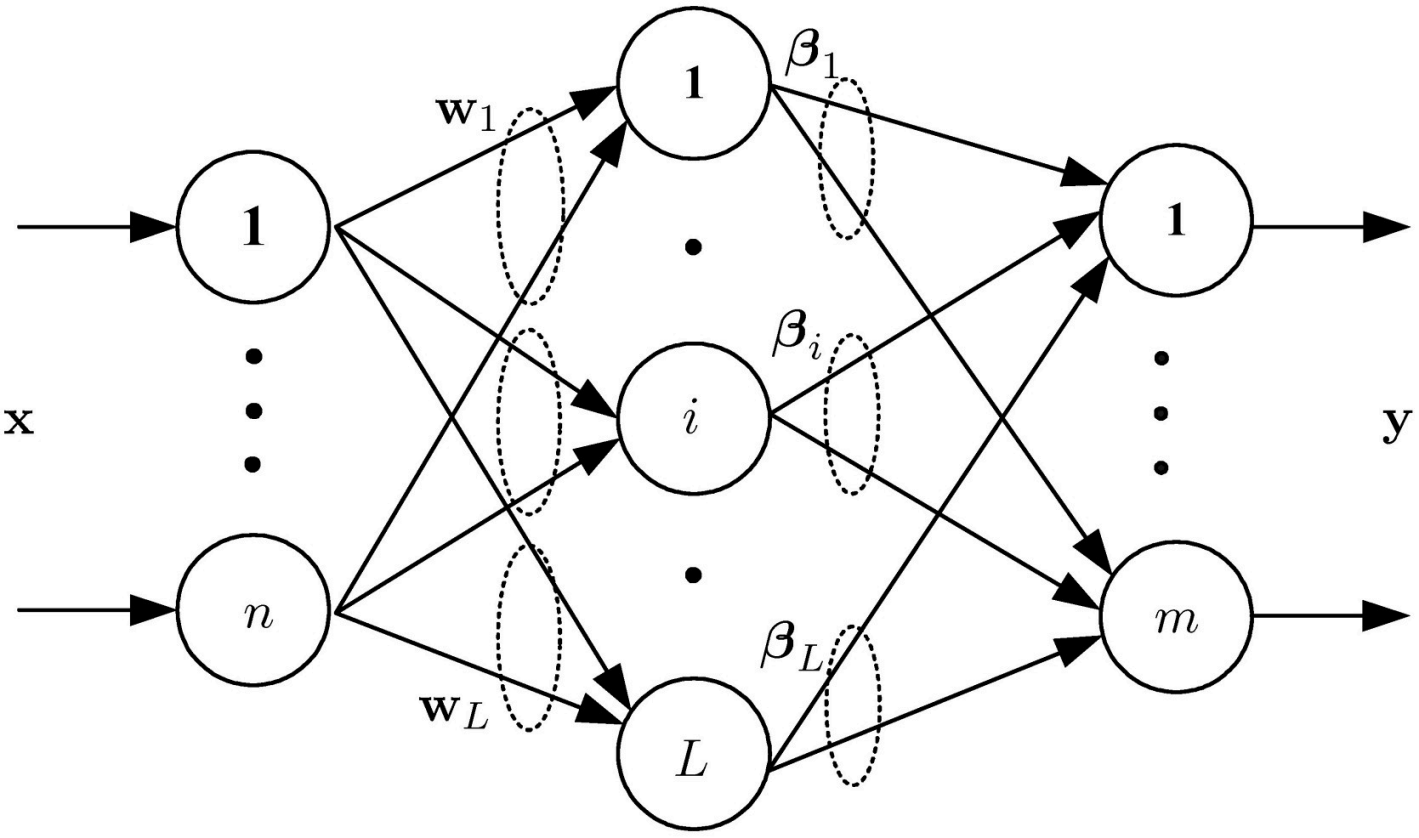
Single-hidden layer feedforward neural network [14].

The typical architecture of a SLFN is shown in Fig 1 [14], in which there are *n* input nodes, *m* output nodes and *L* hidden nodes. Denote $D=\{\left(x_{i},y_{i}\right)|x_{i}\in R^{n},y_{i}\in R^{m},i=1,2,\cdots ,N\}$ be a set of training sample. The output of SLFN is calculated as

$$\begin{array}{c} \sum_{l=1}^{L}\beta_{l}g\left(w_{l}\cdot x_{i}+b_{l}\right)={\hat{y}}_{i},i=1,2,\cdots ,N, \end{array}$$ (6)

where **w**_*l*_ = [*w*_*l*1_, *w*_*l*2_, …, *w*_*ln*_] is called input weight vector, which connects the input nodes and *l*th hidden node; ***β***_*l*_ = [*β*_*l*1_, *β*_*l*2_, …, *β*_*lm*_]^T^ is hidden layer weight vector building link between the *l*th hidden neuron and the output nodes, $b_{l}\in R$ is the bias of the *l*th hidden neuron, **w**_*l*_ ⋅ **x**_*i*_ denotes the inner product between vector **w**_*l*_ and **x**_*i*_, *g*(⋅) is the activation function of hidden layer nodes. In ideal case, one expects the output of SLFN ${\hat{y}}_{i}$ are perfectly equal to the actual output **y**_*i*_, i.e.,

$$\begin{array}{c} \sum_{l=1}^{L}\beta_{l}g\left(w_{l}\cdot x_{i}+b_{l}\right)=y_{i},i=1,2,\cdots ,N. \end{array}$$ (7)

Writing Eq (7) in matrix form, one can get

$$\begin{array}{c} H\beta =Y, \end{array}$$ (8)

where

$$\begin{array}{c} H={\left[\begin{array}{ccc} g(w_{1}x_{1}+b_{1}) & \cdots & g(w_{L}x_{1}+b_{L})\\ \vdots & \cdots & \vdots \\ g(w_{1}x_{N}+b_{1}) & \cdots & g(w_{L}x_{N}+b_{L}) \end{array}\right]}_{N\times L}, \end{array}$$ (9)

$$\begin{array}{c} \beta ={\left[\begin{array}{c} \beta_{1}^{T}\\ \vdots \\ \beta_{L}^{T} \end{array}\right]}_{L\times m}\;\text{and}\;Y={\left[\begin{array}{c} y_{1}^{T}\\ \vdots \\ y_{N}^{T} \end{array}\right]}_{N\times m}. \end{array}$$ (10)

**H** is called the hidden layer output matrix. In Eq (8), the output weights ***β*** is unknown. Prof. Huang [8] proposed to calculate ***β*** using the following M-P pseudo inverse,

$$\begin{array}{c} \beta =H^{†}Y, \end{array}$$ (11)

where **H**^†^ is the Moore-Penrose (M-P) generalized pseudo-inverse of the hidden layer output matrix. The solution presented in Eq (11) means that the smallest error can be obtained.

### *ℓ*_1_-regularized ELM

In practice, determining the number of hidden layer nodes for SLFN is an important problem. If the number of hidden layer nodes is selected too large, over-fitting occurs, and vice versus, if the number of hidden layer nodes is selected too small, under-fitting may occur. In the literatures, some pruning technologies had been proposed to select the appropriate number of hidden layer nodes [15, 16]. Although it has advances in training speed and accuracy, ELM itself can not automatically determine the appropriate number of hidden layer nodes.

It can be seen that the solution of Eq (11) is a least square solution of the following minimization problem,

$$\begin{array}{c} \hat{\beta }=\arg\min_{\beta }{\left\|Y-H\beta \right\|}_{2}^{2}. \end{array}$$ (12)

The least square estimation of output weight ***β*** has smaller variance for training set, however, it has large variance for test set. That is to say, the generalization of ELM is not so satisfactory. On the other hand, least square hasn’t the ability of variable selecting. In ELM, determining the number of hidden layer nodes can be viewed as a problem of variable selecting. Thus, we can add a *ℓ*_1_ penalty term on the output weight as following,

$$\begin{array}{c} \hat{\beta }=\arg\min_{\beta }{\left\|Y-H\beta \right\|}_{2}^{2}+\lambda {\left\|\beta \right\|}_{1}, \end{array}$$ (13)

where the first term ${\left\|Y-H\beta \right\|}_{2}^{2}$ forces the output of ELM is as close as possible to the actual output, the second term λ‖***β***‖_1_ is a *ℓ*_1_-regularized term of output weight ***β*** and λ is the regularization parameter, which is used to perform a tradeoff between the approximation error and the sparsness of the weights. Solving the *ℓ*_1_ regularized problem (13) leads to a sparse solution, i.e., the output weight vector ***β*** is sparse. It means that most of elements of ***β*** are zero or near zero. Therefore, the link between the hidden node and the output is disconnected. The corresponding hidden node can be removed, and thus, the purpose of selecting of the number of hidden nodes is achieved.

There are many methods to solve minimization problem (13), for example, coordinate descent method and gradient descent method. In deed, the problem is also a LASSO (Least absolute shrinkage and selection operator) problem, it can be solved using least angle regression (LAR) algorithm. In this paper, the LAR method is adopted. For more details about LAR, one can refer to [17].

The regularization parameter λ affects the approximation error and the complexity of the model. In most algorithms, the value of λ decrease from λ_*max*_ from λ_*min*_ in a log manner to form a sequence with *K* elements. Each regularization parameter λ(*k*) corresponds to a solution path or a model, in other words. In order to select the best model, several criteria can be adopted. The commonly used criteria includes adjusted *R*^2^, Akaike information criterion (AIC) and Bayesian information criterion (BIC) [18]. In this paper, BIC criteria is used to select the best model. The BIC for *k*-th model is defined as

$$\begin{array}{c} \text{BIC}\left(k\right)={\left\|Y-H\beta \left(k\right)\right\|}_{2}^{2}+\log\left(N\right)M\left(k\right)\sigma \end{array}$$ (14)

where *N* is the number of samples, *M*(*k*) is the number of nonzero elements of ***β***(*k*) and *σ* is the residual variance of a low-bias model defined as

$$\begin{array}{c} \sigma =\frac{1}{N}{\left\|Y-{\text{HH}}^{†}\beta \left(k\right)\right\|}_{2}^{2} \end{array}$$ (15)

where **H**^**†**^ is the Moore–Penrose pseudo-inverse of **H**.

## Ship roll motion prediction based on *ℓ*_1_ regularized ELM

The process of using *ℓ*_1_ regularized ELM for ship roll motion prediction can be summarized as follows.

Step 1 Input the ship roll motion time series {*y*(*k*), *k* = 1, 2, ⋯, *n*};
Step 2 Calculating Lipschitz quotient *q*^(*l*)^ of time series {*y*(*k*)}. From *q*^(*l*)^, *l* = 1, 2, ⋯, determine the input variables.
Step 3 Constructing training set. The input training set is constructed as
  $$X=[\begin{array}{cccc} y(1) & y(2) & \cdots & y(m)\\ y(2) & y(3) & \cdots & y(m+1)\\ \cdots & \cdots & \cdots & \cdots \\ y(n-m) & y(n-m+1) & \cdots & y(n-1) \end{array}],$$
  and the output is as
  $$Y=[\begin{array}{c} y(m+1)\\ y(m+2)\\ \vdots \\ y(n) \end{array}]$$
  where *m* is the number of input variable determined in Step 2. In **X**, each row is a training sample.
Step 4 Training *ℓ*_1_ regularized ELM. Set the hidden layer nodes of ELM to be a large number, and solve the optimization problem (13) using LAR algorithm to obtain *K* models
Step 5 Select the best model using BIC criteria in (14). Let the best model be β*.
Step 6 Prediction using the best model. Let F(⋅) represent the map relationship of the best model. One step ahead prediction is achieved by
  $$\begin{array}{c} \hat{y}\left(k+1\right)=F\left(y\left(k\right),y\left(k-1\right),\cdots ,y\left(k-m-1\right)\right), \end{array}$$ (16)
  and the second step prediction is achieved as
  $$\begin{array}{c} \hat{y}\left(k+2\right)=F\left(\hat{y}\left(k+1\right),y\left(k\right),\cdots ,y\left(k-m-2\right)\right). \end{array}$$ (17)
  *p*-step ahead prediction can be obtained in a similar way as Eqs (16) and (17).

## Simulation studies

**Fig 2:**
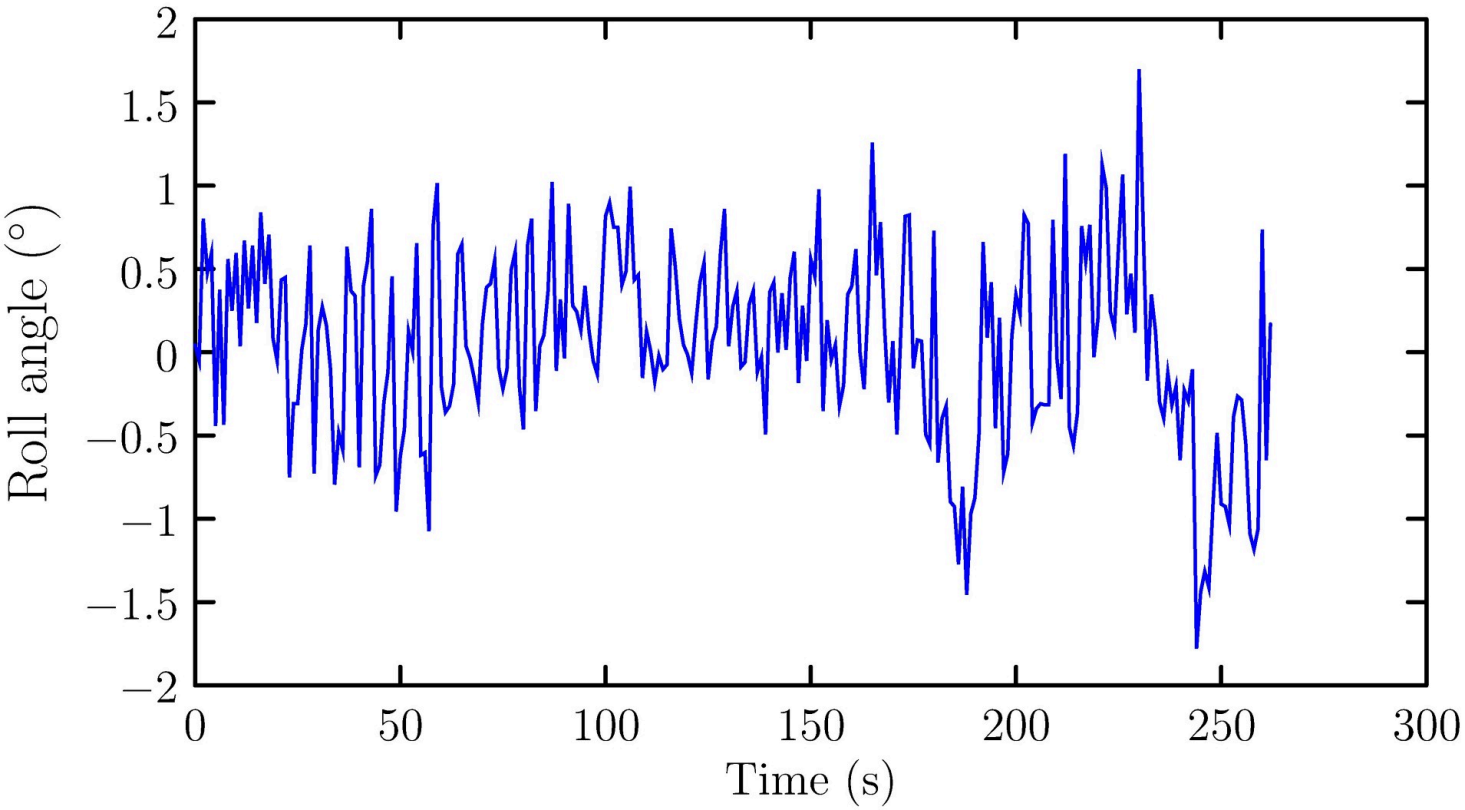
Measured roll angles.

To validate the effectiveness of the proposed ship roll motion prediction method, simulation studies are conducted. All the algorithms in this paper are implemented using MATLAB 2016b programming language and executed on Thinkpad T440, a laptop computer with Intel^®^ Core^™^ i5-4200U processor, 8.0G random access memory (RAM). The roll motion data is measured from *Yu Kun*, a scientific research and training ship. The sea trial condition and characteristic of *Yu Kun*, one can refer to [11]. The measured roll angles are shown in Fig 2.

### Determining the structure of ELM

In this paper, the maximum number of hidden nodes of the ELM is set to 500, i.e., *L* = 500. The activation function is *sigmoidal* function. The training algorithm is LAR algorithm.

**Fig 3:**
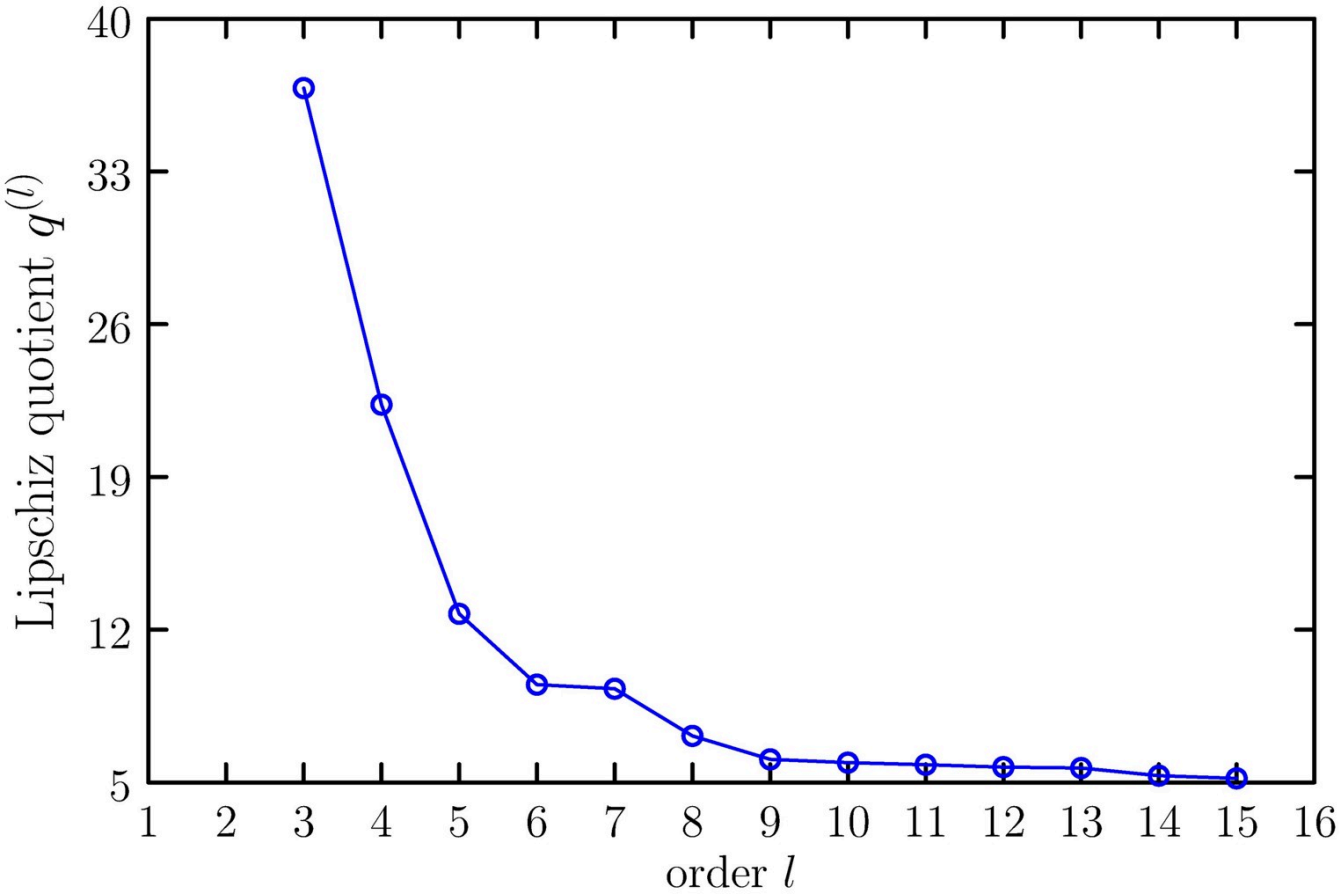
Lipschitz quotients *q*^(*l*)^.

**Fig 4:**
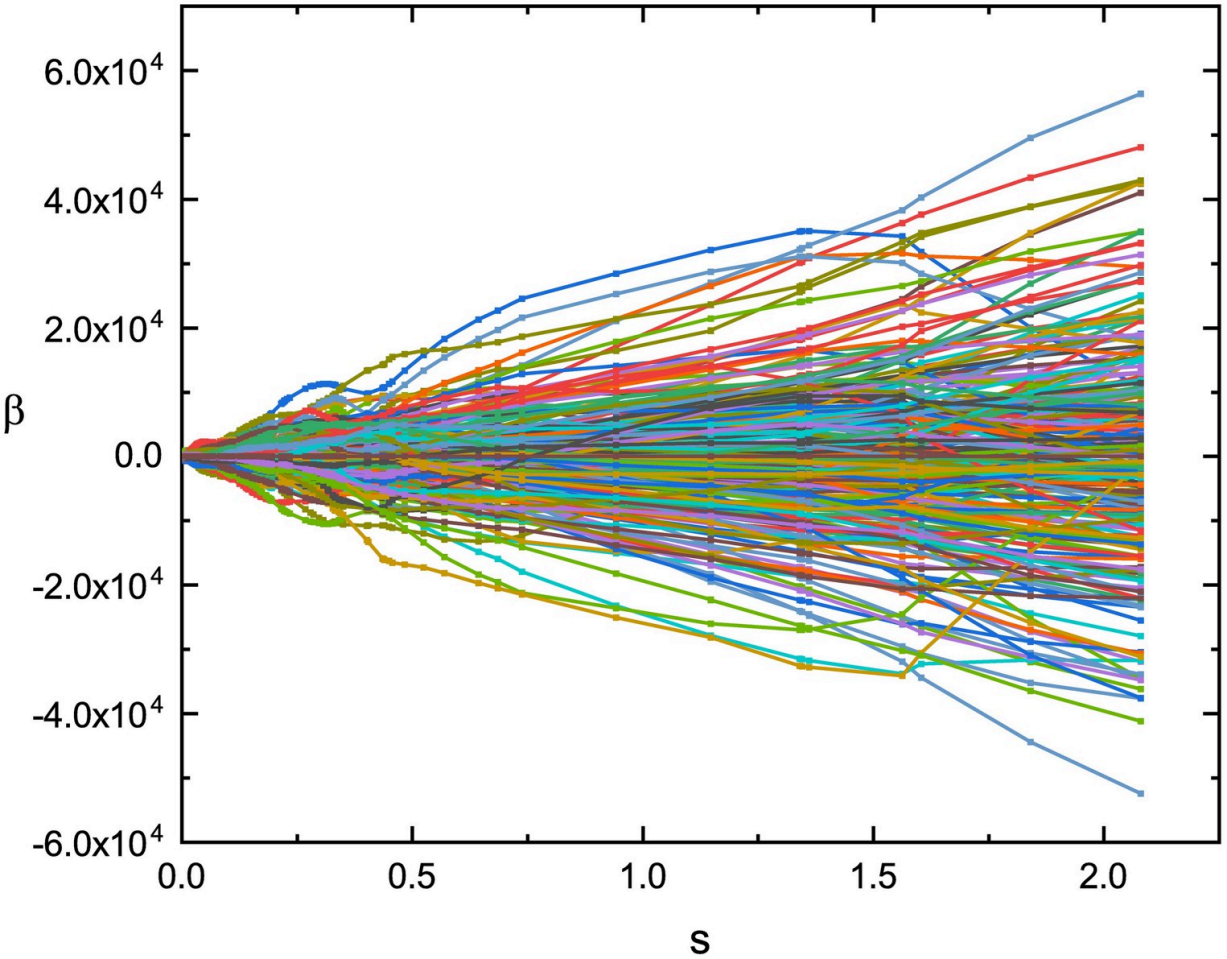
Solution path of LAR.

The Lipschitz quotient method described in section 1 is used to select the input variables of ELM. The Lipschitz quotient *q*^(*l*)^ of measured roll angles is shown in Fig 3. In Fig 3, *q*^(1)^ = *NaN*, *q*^(2)^ = inf, *q*^(3)^ = 36.82, *q*^(4)^ = 22.31, *q*^(5)^ = 12.72, *q*^(6)^ = 9.48, *q*^(7)^ = 9.21. Since, *q*^(6)^ is smaller than *q*^(5)^ and *q*^(6)^ is very close to *q*^(7)^, according to the principle of Lipschitz quotient, the input variables are six, i.e., the input of ELM is {*y*(*k* − 1), *y*(*k* − 2), ⋯, *y*(*k* − 6)}, the output of ELM is *y*(*k*). Therefore, the ELM has initially 6 inputs and 500 hidden nodes and one output. The LAR algorithm is used to training *ℓ*_1_ regularized ELM. Fig 4 shows the solution path of LAR. According to BIC criterion, the finally model contains 256 non-zeros elements in ***β***, i.e., the ELM finally has 256 hidden layer nodes.

### Simulation results

**Fig 5:**
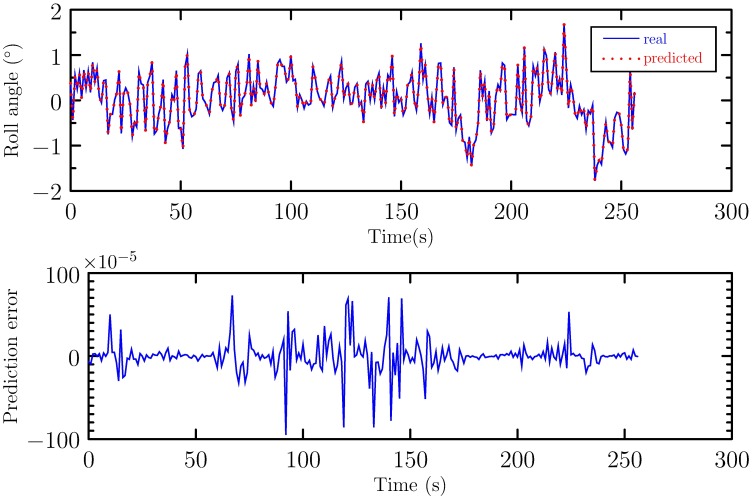
Prediction result and error.

In this section, the simulation results of prediction and prediction performance of the proposed method are presented. The RMSE (root mean square error), defined as

$$\begin{array}{c} \text{RMSE}=\sqrt{\frac{\sum_{k=1}^{N}{\left[y\left(k\right)-\hat{y}\left(k\right)\right]}^{2}}{N}} \end{array}$$ (18)

is used to evaluate the prediction performance. In (18), *y*(*k*) is the real measured roll angle at time *k* and $\hat{y}\left(k\right)$ is the predicted roll angle. The real and the one-step predicted roll angle are shown in Fig 5. Also, the prediction error is shown. It can be seen from Fig 5 that the prediction is accurate with small prediction error. The RMSE of the prediction is 1.9819 × 10^−4^.

**Fig 6:**
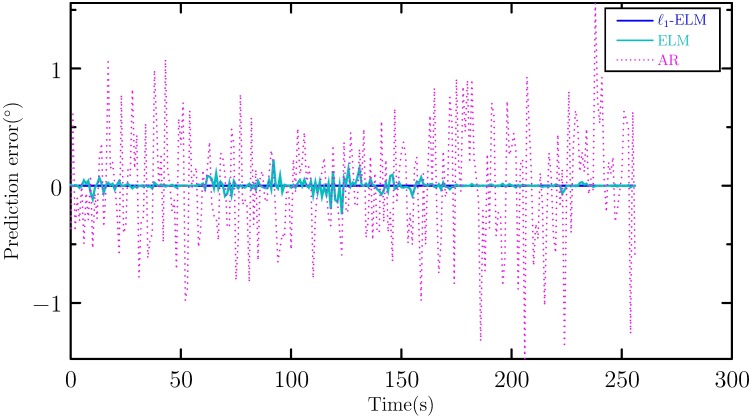
Prediction error of AR, ELM and *ℓ*_1_-ELM.

To show its effectiveness, the proposed method is compared with autoregression (AR) method, conventional ELMs with different inputs and hidden layer nodes. Table 1 lists the comparison results of RMSEs of AR with different number of inputs, ELM with different number of inputs and different number of hidden layer nodes, and *ℓ*_1_-ELM. It can be seen from Table 1 that *with the increase of the number of inputs, the RMSE of AR and ELM decrease, on the other hand, with the increase of the number of hidden layer nodes, the RMSE of ELM also decreases.* This fact implies that selecting the appropriate inputs and hidden layer nodes has great effect on the prediction accuracy. For our proposed method, the inputs and number of hidden layer nodes are objectively determined using suitable algorithms and can obtain more accurate prediction result than conventional ELM and AR methods. This demonstrates the advantage of the proposed method. To show the advantage of the proposed method, the prediction error of AR method with 6 inputs and ELM with 6 inputs and 250 hidden layer nodes are shown in Fig 6.

**Table 1:** RMSEs of different methods.

Inputs—	AR	ELM(# hidden layer nods)					*ℓ*_1_-ELM
		50	100	150	200	250	256
2	0.5031	0.4475	0.4053	0.4044	0.4036	0.4031	-
3	0.5014	0.4487	0.3927	0.3405	0.254	0.1645	-
4	0.4866	0.4371	0.3807	0.2966	0.2049	0.0646	-
5	0.4844	0.4322	0.3855	0.287	0.1819	0.0501	-
6	0.4791	0.4263	0.3663	0.3197	0.2111	0.0422	1.9819 × 10^−4^

## Conclusion

A *ℓ*_1_ regularized ELM based scheme is proposed for ship roll motion prediction. The proposed method combines Lipschitz quotient and *ℓ*_1_ regularized technique to determine appropriate structure of ELM for the purpose of obtaining high accurate prediction. Real measured roll motion data is used to validate the effectiveness of the proposed method. Simulated prediction results show that the proposed method can achieve more accurate prediction than conventional ELM and AR prediction method.
